# ReRouting biomedical innovation: observations from a mapping of the alternative research and development (R&D) landscape

**DOI:** 10.1186/s12992-016-0190-8

**Published:** 2016-09-14

**Authors:** Alexandra Greenberg, Rachel Kiddell-Monroe

**Affiliations:** 1 Universities Allied for Essential Medicines, Washington D.C, United States; 2 Doctors Without Borders, Montreal, Canada

**Keywords:** Global health, Pharmaceutical innovation, Access to medicines, Biomedical research and development (R&D), Intellectual property

## Abstract

In recent years, the world has witnessed the tragic outcomes of multiple global health crises. From Ebola to high prices to antibiotic resistance, these events highlight the fundamental constraints of the current biomedical research and development (R&D) system in responding to patient needs globally.

To mitigate this lack of responsiveness, over 100 self-identified “alternative” R&D initiatives, have emerged in the past 15 years. To begin to make sense of this panoply of initiatives working to overcome the constraints of the current system, UAEM began an extensive, though not comprehensive, mapping of the alternative biomedical R&D landscape. We developed a two phase approach: (1) an investigation, via the RE:Route Mapping, of both existing and proposed initiatives that claim to offer an alternative approach to R&D, and (2) evaluation of those initiatives to determine which are in fact achieving increased access to and innovation in medicines. Through phase 1, the RE:Route Mapping, we examined 81 initiatives that claim to redress the inequity perpetuated by the current system via one of five commonly recognized mechanisms necessary for truly alternative R&D.

Preliminary analysis of phase 1 provides the following conclusions:

1. No initiative presents a completely alternative model of biomedical R&D.
2. The majority of initiatives focus on developing incentives for drug discovery.
3. The majority of initiatives focus on rare diseases or diseases of the poor and marginalized.
4. There is an increasing emphasis on the use of push, pull, pool, collaboration and open mechanisms alongside the concept of delinkage in alternative R&D.
5. There is a trend towards public funding and launching of initiatives by the Global South.

Given the RE:Route Mapping’s inevitable limitations and the assumptions made in its methodology, it is not intended to be the final word on a constantly evolving and complex field; however, its findings are significant. The Mapping’s value lies in its timely and unique insight into the importance of ongoing efforts to develop a new global framework for biomedical R&D. As we progress to phase 2, an evaluation tool for initiatives focused on identifying which approaches have truly achieved increased innovation and access for patients, we aim to demonstrate that there are a handful of initiatives which represent some, but not all, of the building blocks for a new approach to R&D.

Through this mapping and our forthcoming evaluation, UAEM aims to initiate an evidence-based conversation around a truly alternative biomedical R&D model that serves people rather than profits.

## Background: a system putting the profits of a few over the needs of the many

Today the world is witness to the tragic outcomes of the West African Ebola outbreak, the soaring price of medicines in both rich and poor countries, and the lack of major new antibiotics to address the spread of microbial disease. Together these events have further highlighted the constraints of the current biomedical research and development (R&D) system, which make it unresponsive to many of the ever-growing needs of patients globally.

For over two decades, concerns have been raised about the current biomedical R&D system.^1^ Since the passage of international patent protection laws in the mid 1990s [1], evidence of the multiple ways that people in lower income settings are deprived of access to affordable and appropriate medications has grown. The World Health Organization (WHO) has maintained its estimate made in 2000 that at least one in three people worldwide lack access to essential medicines and 10 million people per year die as a result [2]. In 2009, total global investments in health R&D amounted to US$240 billion. Yet in 2010, only about one percent of all health R&D investments were dedicated to neglected diseases [3]. Furthermore, under the current system, therapeutic advances are a rarity with the majority of new drugs showing little to no added value compared to previously available treatments [4].

Today, this crisis of high prices spans the entire disease spectrum and affects populations in all countries. From 2014 to 2015 alone, drug prices increased almost 14 % in the United States (US) [5]. A bipartisan report from the US Senate Finance Committee showed that Gilead’s “pricing strategy was focused on maximizing revenue” [6]. The result is a price tag of US$84,000 for a 12-week course of their Hepatitis C treatment Sofosbuvir, brand-named Sovaldi ® [7]. A recent study showed that manufacturing costs for similar drugs are as low as US$21 to US$63 per 12-week treatment course [8]. A study on the pricing of four oral cancer drugs found that mass generic production of the evaluated drugs could result in treatment prices ranging from US$128 to $4020 while current US prices range from $75,161 to $139,138 [9]. The true cost of developing a novel drug varies depending on whom you ask. Industry claims that high prices are justified in order to recoup the alleged massive investment required to complete R&D. A widely refuted report from Tufts university claims it costs approximately $2.6 billion per drug [10, 11]. However, product development organizations, among others, have quoted far lower values. The Drugs for Neglected Diseases Initiative (DND*i*), for example, estimates the cost of drug development to be $110 million [12]. Additionally, civil society groups and academics have clear analysis demonstrating how drug prices often have little to do with the actual cost of R&D [13]. As the US Senate Finance Committee pointed out in its recent investigation, drug prices have more to do with the price that the market will bear [14]. This market logic guiding corporate pricing is also reflected in the pharmaceutical industry’s neglect of many unprofitable areas of R&D.

## We need a people-centered framework for R&D

On the surface, the UAEM Re:Route Mapping shows an impressive range of alternative biomedical R&D initiatives challenging the current system by tackling issues that have long contributed to lack of access to and innovation in medicines. Furthermore, the implementation of delinkage, wherein the price of a medicine is not correlated with the cost of its R&D, by many of the initiatives indicates a step in a positive direction. Yet, initial observations from the Mapping point to a lack of fundamental systemic change. While some initiatives have undoubtedly made important advances on specific diseases and broader systemic issues, others are simply promoting a “business as usual” approach. And despite these manifold initiatives claiming to fix the system, shockingly little has changed for the majority of people trying to access essential medicines. The Ebola outbreak epitomizes the pitfalls of the current piece-by-piece approach. GAVI’s recent commitment to further develop, license and stockpile an Ebola vaccine previously owned by Merck & Co Inc. is laudable [15]. However, it comes too late for many. The West African Ebola outbreak has claimed over 11,000 lives [16] while GlaxoSmithKline and the National Institutes for Health left the vaccine sitting on the shelf for years due to lack of potential profits from its further development [17]. If a system that worked to meet the needs of all populations had been in place, a vaccine for Ebola, and now for Zika virus, would already exist and be on the market before these diseases became a global threat [18].

The Mapping supports ongoing calls for a new approach to R&D. Over the past decade, various actors and policy processes have acknowledged the need for a new approach to medical innovation. One key proposal is to create a global agreement or framework for biomedical R&D.^1^ The need for new global approaches was reinforced by United Nations (UN) Secretary-General Ban ki-Moon when he called for a new deal on access to medicines during the establishment of the High-Level Panel for Access to Medicines in 2015 [19]. The Mapping reveals a fragmented and haphazard landscape that will not lead to a truly new approach to biomedical R&D. One reason is that many initiatives are based on the notion that the current biomedical R&D system needs to be fixed and some of its side effects remedied. Yet, the current system is not broken. It was designed to treat health as a commodity and it is successfully maintaining that approach. The myriads of short and long-term fixes currently being debated are important steps in the process but will not change the fundamental nature of that profit-driven system [20]. In our view, while incremental steps are important and necessary, it is past time to directly address the underlying constraints inherent in the current R&D system.

## Mapping alternatives: methodology, limitations & preliminary observations

### Methodology

A large and growing number of self-identified “alternative” R&D initiatives have emerged in the past fifteen years [20]. In light of the 2005 WHO Commission on Intellectual Property Rights, Innovation and Public Health (CIPIH) Report, the WHO Consultative Expert Working Group on Research and Development: Coordination and Financing (CEWG) actively called for projects to demonstrate alternative ways of approaching biomedical R&D and since then the alternative landscape has continued to evolve [21]. In order to understand which of the multiple initiatives truly respond to the ongoing call for alternatives, Universities Allied For Essential Medicines (UAEM) carried out an extensive, though not exhaustive, two phase mapping of the alternative biomedical R&D landscape. Phase 1 mapped the initiatives, existing or proposed, that claim to be an alternative approach to biomedical R&D. Phase 2, not yet published, will evaluate which of those existing and proposed initiatives are already achieving increased access to and innovation in medicines.

The Re:Route Mapping represents phase 1 of this effort. We reviewed more than 130 “alternative” initiatives using a simple methodology based on a specific set of defined inclusion and exclusion criteria. These were developed from accepted alternative mechanisms [22] such as delinkage of prices from costs of R&D, openness, collaboration and use of push, pull, and/or pooling (Refer to Criteria). We retained 81 initiatives that aim to redress the shortcomings of the current biomedical R&D system and that fulfilled at least one of the inclusion criteria. We then separated the initiatives into existing initiatives (49), which are currently underway, and proposed initiatives (32), which are not yet in place but show signs of ongoing development. We categorized the initiatives by first separating them according to the stage of the biomedical R&D system they primarily seek to address, and second identifying the key innovation and access principles they apply. This allowed us to identify types of initiatives through mode of action within the R&D process and then to further differentiate each initiative in terms of alternative mechanisms to financing and/or completing R&D that are regularly implemented.

## Criteria: alternative mechanisms for biomedical R&D [23]

- *Push Mechanism*: Direct funding for R&D, often in the form of a grant, as well as indirect incentives, such as tax breaks and in-kind contributions, which help finance R&D upfront and thus mitigate the R&D investment required.
- *Pull Mechanism*: Mechanisms to incentivize R&D activities through the promise of financial rewards once specified objectives or milestones have been met, creating viable market demand.
- *Pooling Mechanism*: Pooling of funds that are aggregated and managed jointly by an established entity to be allocated based on priority setting in order to distribute risk and finance biomedical R&D. Additionally, pooling of intellectual property (IP), typically via a patent pool, an agreement between two or more patent owners to pool their patent rights and license the rights to use these patents together to one another as well as third parties. These two distinct types of pooling can occur independently or jointly.
- *Collaborative Initiative*: Involves a network, consortium, or partnership between two or more of any academic or research institutions, non-profit organizations, non-governmental organizations (NGOs), governments, government entities, or members of the private sector including biotech and pharmaceutical companies and is often used to facilitate knowledge sharing.
- *Open Initiative*: Applies open source, open access, open data, or open knowledge principles. Interested parties are able to contribute knowledge or know-how, data, technology, etc. to be shared in the public domain and, in the case of open source, in coordination with patent-free research.

### Limitations

We recognize that the Mapping methodology has limitations and that there are gaps. From the outset, our approach was driven by the pressing need to develop a consolidated “snapshot” of the current alternative R&D landscape, especially given the various high level international processes undertaken to better understand and address the constraints of the current system [24] This project was not meant to result in a comprehensive and objective scientific study of alternative approaches. Given the rapidly developing context and the lack of clarity around what is or is not alternative, we were forced to make assumptions. However, several experts reviewed our methodology and, while there are potentially other ways to categorize alternative biomedical R&D initiatives, together we agreed that separating initiatives by phase of the pipeline was the least subjective of the typologies considered.

There is currently no existing basis for categorization of these initiatives so we had to determine what methodology was most appropriate as well as apply it using peer review and consensus building. We accept that some initiatives could be classified differently. Similarly, some overlap and blurring between categories and approaches is inevitable. We used our best judgment based on the information publicly available to us. We provide an extensive glossary in the Mapping to explain our approach and assumptions. In the spirit of openness and collaboration, we are open to feedback and critique.

As set out in the important user notes of the Mapping, this Report does not aim to make any judgments on the value of different areas of drug development or individual initiatives and their work. Rather, it highlights areas where alternative approaches are currently more commonly applied. The report draws attention to what is already out there and provides a starting point for an ongoing and inclusive dialogue informed by facts. It presents reliable and significant preliminary findings, summarized below, and indicates that more research needs to be done in order to better understand how to unify and coordinate efforts towards needs-driven R&D.

### Preliminary observations

**Fig. 1:**
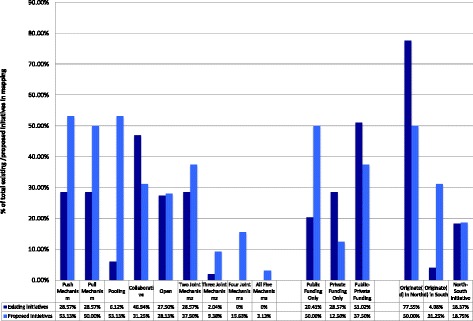
Preliminary Findings. The figure shows the breakdown of included initiatives based on whether they implemented one or more of the defined alternative mechanisms as well as based on source of funding and geographical origin

**Firstly, none of the initiatives included in the Mapping present a completely alternative model of biomedical R&D or a new system** (Fig. 1). Having classified the initiatives according to elements of the biomedical R&D process, we see the majority of the initiatives address just one aspect of the biomedical R&D chain, a few try to address multiple parts of the pipeline and very few seek to apply a revolutionary or novel approach to biomedical R&D.

Among the 49 existing R&D-related initiatives, we did not find an initiative that both addressed four or more of the five key innovative R&D mechanisms we identified (push, pull, pool, collaborative and open) and utilized the concept of delinkage. Of the 32 proposed initiatives however, we found six (19 %) that covered four or more key mechanisms and embraced the concept of delinkage. Disappointingly, these six initiatives remain un(der)funded and/or without active projects.

**Second, the initiatives are largely focused on rare diseases or diseases of the poor and marginalized.** Nearly 9 in 10 of the existing initiatives focus on neglected tropical diseases (NTDs), rare diseases or malaria, Tuberculosis (TB) and/or HIV, while 40 % (19) of the proposed initiatives focus on NTDs and rare diseases. While NTDs represent real needs, the fact that only 3 existing initiatives and 1 proposed initiative focus on chronic and non-communicable diseases (NCDs) highlights a lack of attention to an important and distinct portion of global health needs [25]. New antibiotic development is a key focus of only 2 % of existing but 16 % of proposed initiatives, all of which are based in the North and publicly funded. It is also striking to note that only 4 % (2) of the existing initiatives and 32 % (7) of the proposed initiatives focus on diagnostics, a critical stage of medical treatment.

**Third, there is an increasing emphasis on the use of push, pull, pool, collaboration and open mechanisms alongside the concept of delinkage in alternative R&D** (Fig. 1). The number of initiatives implementing push mechanisms has increased from 14 % existing (7) to 50 % proposed (16), the number of initiatives with pooling has risen from 2 % existing (1) to 47 % proposed (15) and openness is an overt strategy in 10 % of existing (5) compared to 32 % of proposed (10) initiatives. Pull mechanisms have become slightly more prevalent, present in 16 % of existing (8) and 25 % of proposed (8) initiatives, whereas collaboration has remained fairly constant, present in 24 % of existing (12) and 28 % of proposed (9) initiatives. Additionally, implementation of more than two mechanisms is prevalent in both existing (32.5 %) and proposed (56 %) initiatives. While only one existing initiative applies three mechanisms together, 8 proposed initiatives apply three or more mechanisms, indicating an increase in joint application of alternative mechanisms.

**Finally, we observed that there is a trend towards public funding and launching of initiatives by the Global South** (Fig. 1). Only 20 % of existing (10) initiatives rely solely on public funding sources, compared to 50 % of proposed (16) initiatives. As demand for public funding has grown, reliance solely on private funding sources has dropped from 29 % existing to 12 % proposed. Reliance on public and private funding sources together has also declined from 51 % existing to 38 % proposed. While, 78 % of existing and 69 % of proposed initiatives originated in the North, there seems to be a slight trend towards more initiatives being developed in the South.

## Next steps

In light of these findings, UAEM is now launching the second phase of the mapping process, the development of an evaluation tool for initiatives, focused on identifying which practices or approaches have achieved increased pharmaceutical innovation and access to medicines for patients. Further analysis on aspects of included initiatives such as funding, intellectual property, and pricing will be conducted after we have collected additional data to xmake our evaluation as robust and informative as possible. UAEM believes that through collaborative, criteria-based and in-depth evaluation, the practices applied and principles followed by promising initiatives can serve as building blocks for an overarching framework to govern and guide R&D. This tool will be available online and will evaluate and score all 81 initiatives in key metrics relating to access, innovation, and collaboration. It will be modeled after UAEM’s Report Card project, making it feasible to directly compare initiatives and provide key evidence concerning best practices as well as where improvement remains needed in alternative R&D. Publicly sharing this information through an online evaluation tool will foster renewed and ongoing dialogue concerning the criteria and principles to be laid out for a framework for biomedical R&D that is patient-oriented and needs-driven.

## Conclusion

While we are morally bound to do what we can today to improve patient access to medicines, we must also be bold enough to collectively aim for and work towards a novel, ethical and rights-based way of carrying out biomedical R&D that can benefit all those who need access to lifesaving medicines. Through this mapping and through the evaluation of existing and proposed alternative R&D initiatives, UAEM aims to initiate an evidence-based conversation around a truly alternative biomedical R&D model that serves people rather than profits.

To view UAEM’s Re:Route Mapping, please visit www.altreroute.com

## Abbreviations

CEWG: Consultative Expert Working
CIPIH: Commission on Intellectual Property Rights, Innovation and Public Health
DND*i*: Drugs for Neglected Diseases Initiative
NCD: Non-communicable Disease
NGO: Non-governmental Organization
NTD: Neglected Tropical Diseases
R&D: Research and Development
TB: Tuberculosis
UAEM: Universities Allied for Essential Medicines
UN: United Nations
US: United States
WHO: World Health Organization

## Footnotes

1 **Actors and stakeholders recommending new global rules for R&D include**: (1) The United Nations Secretary-General’s High-Level Panel (UNHLP, 2015) on Access to Medicines, info available at http://www.unsgaccessmeds.org/, (2) Suerie Moon, Jorge Bermudez, and Ellen’t Hoen. “Innovation and access to medicines for neglected populations: could a treaty address a broken pharmaceutical R&D system?” PLoS medicine 9.5 (2012): 522., (3) Health Action International Global, Initiative for Health and Equity in Society, Knowledge Ecology International, Medecins Sans Frontieres,Third World Network (2011) An essential health & biomedical treaty. Available at http://www.who.int/phi/news/phi_1_joint_submission_en.pdf., (4) WHO Consultative Expert Working Group on Research and Development: Financing and Coordination (CEWG, 2009) & Demonstration Projects, info available at http://www.who.int/phi/cewg/en/, (5) Governments of Bangladesh, Barbados, Bolivia, and Suriname (2009) Proposal for WHO discussions on a biomedical R&D treaty. Available at http://www.who.int/phi/Bangladesh_Barbados_Bolivia_Suriname_R_DTreaty.pdf (6) Intergovernmental Working Group (IGWG, 2006) on Public Health, Innovation and Intellectual Property & Global Strategy and Plan of Action (GSPoA, 2008) for Public Health Innovation and Intellectual Property. Available at http://www.who.int/phi/igwg/en/, (7) Dentico N, Ford N (2005) The courage to change the rules: A proposal for an essential health R&D treaty. PLoS Med 2(2): e14. doi:10.1371/journal.pmed.0020014., (8) Hubbard T, Love J (2004) A new trade framework for global healthcare R&D. PLoS Biol 2(2): e52. doi:10.1371/journal.pbio.0020052., and (9) WHO Commission on Intellectual Property Rights, Innovation and Public Health (CIPIH, 2003). Available at http://www.who.int/intellectualproperty/en/
